# Spatiotemporal Dynamics of the Carbon Budget and the Response to Grazing in Qinghai Grasslands

**DOI:** 10.3389/fpls.2021.775015

**Published:** 2022-01-07

**Authors:** Xiaotao Huang, Chunbo Chen, Buqing Yao, Zhen Ma, Huakun Zhou

**Affiliations:** ^1^Key Laboratory of Restoration Ecology for Cold Regions Laboratory in Qinghai, Northwest Institute of Plateau Biology, Chinese Academy of Sciences, Xining, China; ^2^Key Laboratory of Adaptation and Evolution of Plateau Biota, Chinese Academy of Sciences, Xining, China; ^3^University of the Chinese Academy of Sciences, Beijing, China; ^4^State Key Laboratory of Desert and Oasis Ecology, Xinjiang Institute of Ecology and Geography, Chinese Academy of Sciences, Urumqi, China

**Keywords:** spatiotemporal dynamics, gross primary productivity, net ecosystem exchange, grazing, model

## Abstract

Estimating the grassland carbon budget is critically important for ensuring that grassland resources are used sustainably. However, the spatiotemporal dynamics of the carbon budget and the response to grazing have not yet been characterized in Qinghai grasslands. Here, we estimated the gross primary productivity (GPP) and net ecosystem exchange (NEE) in Qinghai grasslands using the improved Biome-BGCMuSo model to characterize the spatiotemporal dynamics of the carbon budget and the response to grazing in this region from 1979 to 2018. The GPP of Qinghai grasslands fluctuated during the study period, with an average annual value of 118.78 gC/m^2^. The NEE of Qinghai grasslands fluctuated from 1979 to 2018, with an average value of −5.16 gC/m^2^. After 2,000, GPP increased, and NEE decreased in a fluctuating manner. There were clear regional differences in GPP and NEE. GPP was low in most areas of Qinghai, and GPP was high in eastern and southern Qinghai. The southern, southeastern, and northeastern parts of Qinghai were mainly carbon sinks, and the northwestern part of Qinghai and the region between the southeastern and northeastern parts of Qinghai were mainly carbon sources. Grazing generally decreased GPP and increased NEE in Qinghai grasslands from 1979 to 2018. There was spatial heterogeneity in the effect of grazing on GPP and NEE. Under grazing, GPP and NEE were significantly decreased mainly in eastern Qinghai, and GPP and NEE were significantly increased mainly in southern and eastern Qinghai. NEE was most affected by grazing in eastern Qinghai. The results of this study aid our understanding of the mechanism driving variation in the grassland carbon budget and provide new data that could be used to support local grassland management.

## Introduction

Quantitative assessment of the carbon budget of terrestrial ecosystems is essential for regional carbon sink management and the implementation of action plans for climate change mitigation (Webb et al., 2019; Giltrap et al., 2020; Li et al., 2020). Assessment of the spatiotemporal dynamics of the terrestrial ecosystem carbon budget is not only an important goal of research on ecosystems and global change but is also key for achieving objectives set by the United Nations Framework Convention on climate change and global greenhouse gas management (Ford et al., 2016; Bakhtiari, 2018; Qiu et al., 2021). Gross primary productivity (GPP) is the amount of CO_2_ that is taken up by plants from the atmosphere through photosynthesis (Chai et al., 2017), and it directly reflects the photosynthetic capacity of plants (Zhu et al., 2016; Liu et al., 2021). The net ecosystem exchange (NEE) is the difference between GPP and the efflux of CO_2_ released back to the atmosphere through ecosystem respiration processes (Hao et al., 2020; Nascimento Alves et al., 2021). It directly reflects the carbon sink capacity of ecosystems (Yang et al., 2019). They are both key indicators of the terrestrial carbon budget. Accurate estimation of GPP and NEE is important for determining the global carbon budget. Grassland ecosystems are an important part of terrestrial ecosystems. In addition to providing high-quality forage and livestock products, conserving water and soil, and maintaining biodiversity, they also absorb CO_2_ from the atmosphere through photosynthesis and play a key role in the carbon cycle. The grassland carbon budget depends on climate change and management. Several studies have examined the carbon budget of grassland ecosystems at different scales (Chai et al., 2017; Wang et al., 2019; Li et al., 2020; Song et al., 2020). These previous studies have shown that estimates of the carbon budget can vary greatly among regions and depend on the methods used and the grassland types analyzed (Gilmanov et al., 2007; Moinet et al., 2017; Rong et al., 2017; Zhao et al., 2019; Song et al., 2020; You et al., 2020).

Qinghai is located in the northeastern part of the Qinghai-Tibet Plateau, which is known as “the roof of the world.” It is the site of origin of the Yellow River, Yangtze River, and Lancang River, and it has high ecological significance (Schaller et al., 1988; Gao et al., 2017; Dong Y. et al., 2020). Grassland is the most widely distributed ecosystem in this region. It greatly affects the carbon balance of the regional ecosystem and is the largest carbon pool among the terrestrial ecosystems of Qinghai (Liu et al., 2018; Song et al., 2020). Because of the harsh climate and environment in this region, the ecosystem is extremely fragile and highly sensitive to climate change and human activities (Dong et al., 2015; Sun et al., 2019). Grazing is the most important form of land use of the grasslands in this region, and grassland degradation is widespread because of excessive grazing. Grazing directly affects the grassland carbon budget and thus the sustainable utilization of local grassland resources (Dong et al., 2015; Abdalla et al., 2018; Dong S. K. et al., 2020). Studying the effects of climate change and grazing on the carbon budget in Qinghai grasslands is important for characterizing the pathways and mechanisms driving the grassland carbon cycle. Several studies have examined the carbon budget in Qinghai grasslands (Peng et al., 2017; Sun et al., 2019; Xu et al., 2019; Song et al., 2020). However, previous studies have been conducted at single sites. Consequently, our understanding of the spatiotemporal dynamics of the grassland carbon budget and the response to grazing in Qinghai is poor in light of heterogeneity in terrain, climate, and grazing, and this impedes the sustainable utilization of local grassland resources.

The effect of grazing on the carbon budget of grassland ecosystems has mainly been evaluated through experimental observations, remote sensing, and model simulation (Lin et al., 2017; Jia et al., 2018; Ford et al., 2021; Li et al., 2021). Experimental observations are often too time-consuming and labor-intensive to be conducted over large areas, especially when there is high spatial heterogeneity in climate, terrain, and management regimes. In addition, humans are barred from setting foot in large areas of the grasslands in Qinghai. Thus, a robust understanding of the spatiotemporal dynamics of the grassland carbon budget cannot be feasibly achieved *via* experimental observations. Although remote sensing can be used over large areas, the response of grassland ecosystems to grazing cannot be determined because remote sensing images only provide insight into real scenarios. Model simulation combined with observational data is considered the most effective method for studying the spatiotemporal dynamics of the grassland carbon budget and the response to grazing over large areas because it allows different scenarios (e.g., grazed and ungrazed scenarios) to be evaluated in the same region *via* model estimation (Anh Nguyet and Kawasaki, 2017; Sanchez-Ruiz et al., 2018; Tian et al., 2020). Observational data can then be used to validate the reliability of model outputs. The Biome-BGC model, which is a process-based model, has been used to study the carbon cycle in many regions such as the Qinghai-Tibet Plateau (Bond-Lamberty et al., 2007; Hidy et al., 2012; Mao et al., 2017; You et al., 2019). Hidy et al. (2016) developed the Biome-BGCMuSo model, which is based on the Biome-BGC model. This new model can better simulate the carbon budget of grassland ecosystems than the Biome-BGC model, especially in areas under energy and water stress. In Qinghai, plant growth is mainly limited by energy because of the alpine environment (Shi et al., 2018; Sun et al., 2019). Arid and semi-arid grasslands are widely distributed in this region (Shi et al., 2018; Niu et al., 2021). Thus, the Biome-BGCMuSo model is more applicable to Qinghai than the Biome-BGC model. In addition, the effect of the grazing process is also included in the Biome-BGCMuSo model. The disadvantage of the Biome-BGCMuSo model is that it can only be used for site simulation, which limits its application over large areas (Hidy et al., 2016).

The aim of this study was to examine the spatiotemporal dynamics of the carbon budget and the response to grazing in Qinghai. Therefore, we developed a spatially explicit Biome-BGCMuSo model and used the improved Biome-BGCMuSo model combined with field data from Qinghai grasslands to (1) simulate and analyze the spatial and temporal dynamics of GPP and NEE under climate change and (2) quantify the effect of grazing on GPP and NEE in Qinghai grasslands from 1979 to 2018.

## Materials and Methods

### Study Area

**FIGURE 1:**
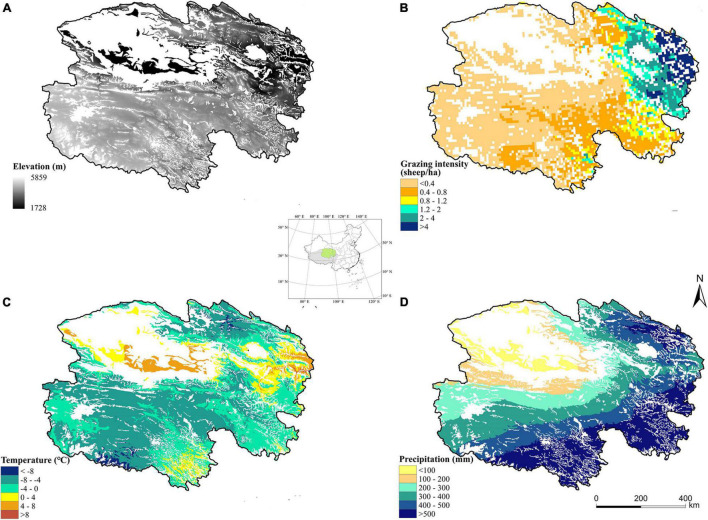
Elevation **(A)**, average annual grazing intensity **(B)**, average annual temperature **(C)**, and average annual precipitation **(D)** in Qinghai grasslands from 1979 to 2018.

Qinghai is located in northwestern China and the northeastern part of the Qinghai-Tibet Plateau (31°39′–39°19′N, 89°35′–103°04′E). Mountains are numerous, and the terrain is diverse. The average altitude is over 3,000 m. The region features a plateau continental climate. The summers are short, and the annual average temperature ranges from −6 to 9°C. The average annual precipitation is between 250 and 550 mm. Qinghai has the largest range in precipitation among all provinces in China. Qinghai is one of the main pastoral areas in China. The natural grassland area (4.19 × 10^7^ hm^2^) accounts for 10.72% of the total grassland area in China, and the available grassland area is approximately 3.87 × 10^7^ hm^2^. The grasslands are mainly distributed in the Qilian Mountains, Qingnan Plateau, and Qaidam Basin. The ecological environment in this region is sensitive to climate change and human activities. Grassland degradation was previously widespread in Qinghai because of unrestricted grazing activities (Figure 1; Gao et al., 2017; Zhao et al., 2020).

### Methods

In the Biome-BGCMuSo model, GPP was estimated using Farquhar’s photosynthesis routine (Farquhar et al., 1980) and the enzyme kinetics model (Woodrow and Berry, 1988). NEE was calculated as follows:


(1) NEE=GPP-mr-gr-hr-dr

where, mr is maintenance respiration, gr is growth respiration, hr is heterotrophic respiration, and dr is the defoliation rate.

The parameters used in this study were based on default values for C_3_ grass (Hidy et al., 2016; You et al., 2019). Some parameters were corrected based on data collected from local investigations. The simulation process was divided into two stages. The first stage was the spinup simulation, which estimates the initial values of the carbon and nitrogen pools. The second stage is the normal simulation, which uses the results of the spinup simulation as initial values for the state variable and generates estimates of GPP and NEE in the Qinghai grassland ecosystem.

The R package RBBGCMuso^1^ supports the application of the Biome-BGCMuSo model in the R environment. This means that we can run the Biome-BGCMuSo model using RBBGCMuso. To apply the Biome-BGCMuSo model over a large area, a loop program was designed to iteratively run the RBBGCMuso model; the Biome-BGCMuSo model can be run at different sites when running the loop program. To obtain GPP and NEE over large areas, we divided the study area into grids with a 10 × 10 km resolution, and a loop program was designed to run the Biome-BGCMuSo model in different pixels of Qinghai grasslands. To analyze the effect of grazing on the carbon budget, two scenarios were designed in this study: climate change and climate change combined with grazing. The effect of grazing on the carbon budget was determined by taking the difference between the two scenarios.

### Data

#### Meteorological Data

Data from 1979 to 2018 for precipitation, temperature, saturated vapor pressure difference, day length, and solar radiation were input into the model in daily steps. These data were extracted from the China meteorological forcing dataset by programming. This dataset was based on existing international Princeton reanalysis data, GLDAS data, GEWEX-SRB radiation data, and TRMM Precipitation Data as the background field and integrated with conventional meteorological observation data of the China Meteorological Administration. The spatial resolution was 10 km × 10 km. Comparison of this dataset with the observed data (including station observations in Qinghai) by the producer revealed that the dataset was accurate and superior to the existing reanalysis data (He et al., 2020; Yao et al., 2021).

#### Grazing Data

The grazing intensity data for the year 2010 were derived from global livestock information system data, which were generated by the Food and Agriculture Organization of the United Nations (FAO) by interpolation according to the global livestock number at sampling points and environmental data. The gridded livestock data were presented at a spatial resolution of 0.083333 decimal degrees (approximately 10 km at the equator) in the raw data.^2^ We resampled these data to 10 km × 10 km so that they matched the spatial resolution of meteorological data from the China meteorological forcing dataset. To produce time series grazing data and ensure high precision, we corrected the data using livestock numbers for the year 2010 for different regions and then conducted linear interpolation using livestock statistics from 1979 to 2018 for different regions. The livestock statistics were obtained from the local government. The grazing calendar was set according to a local survey. In this study, we used sheep units to integrate data from different types of livestock. One yak equals 4.5 sheep, one cow equals 6.0 sheep, one goat equals 0.9 sheep, one horse equals 6.0 sheep, and one camel equals 8.0 sheep. These conversion coefficients were determined by the Ministry of Agriculture of the People’s Republic of China^3^ and a survey of local herdsmen.

#### Observational Data

Observational data included GPP and NEE. These data were point-based and collected from previous studies to validate the outputs of the Biome-BGCMuSo model. GPP data were obtained from Maqin County for the year 2006, Tongde County for the years 2012–2016, Golmud for the years 2015–2016 and 2019, Menyuan County for the years 2002–2016, and Qilian County for the years 2002–2003, 2006–2009, 2013, and 2015–2016. Three sites were grazed grasslands, and six sites were ungrazed grasslands (Wu et al., 2010; Sun et al., 2019; Xu et al., 2019; Song et al., 2020; Wang et al., 2020). NEE data were obtained from Maqin County for the years 2006 and 2008, Qilian County for the years 2013, 2015, and 2016, Tongde County for the years 2012–2016, Gonghe County for the year 2015, Golmud for the years 2015–2016 and 2019, Gangcha County for the years 2014–2015, Haiyan County for the years 2010–2011, and Menyuan County for the years 2002–2016. Eight sites were grazed grasslands, and seven sites were ungrazed grasslands (Wu et al., 2010, 2018; Zhang et al., 2012; Peng et al., 2017; Wang et al., 2017, 2020; Chai et al., 2018; He et al., 2019; Li et al., 2019; Sun et al., 2019; Song et al., 2020; Zhu et al., 2021). Observed NEE data in Menyuan County for the years 2014 to 2015 were collected under both the grazed and ungrazed scenarios at the same site (Li et al., 2019).

#### Other Auxiliary Data

Other auxiliary data included elevation data and soil data. Elevation data were obtained from the Geographic Data Sharing Infrastructure, College of Urban and Environmental Science, Peking University.^4^ Soil data, including texture and pH, were obtained from the Harmonized World Soil Database (HWSD)^5^.

## Results

### Model Validation

**FIGURE 2:**
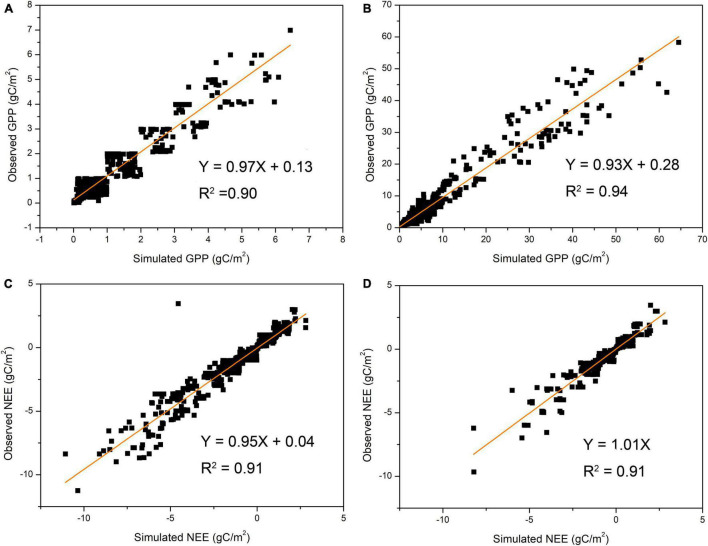
Comparison of GPP values based on simulated and observational data under grazed **(A)** and ungrazed **(B)** conditions and NEE values under grazed **(C)** and ungrazed **(D)** conditions (GPP, gross primary productivity, NEE, net ecosystem exchange).

In this study, the reliability of the model outputs was validated by comparing simulated results with observed data. Figures 2A,B shows that the Biome-BGCMuSo model performed well in predicting GPP under both the grazed (*R*^2^ = 0.90′) and ungrazed grasslands (*R*^2^ = 0.94). Figures 2C,D shows that the Biome-BGCMuSo model performed well in predicting NEE under both the grazed (*R*^2^ = 0.91) and ungrazed grasslands (*R*^2^ = 0.91).

### Temporal and Spatial Dynamics of Gross Primary Productivity and Net Ecosystem Exchange

**FIGURE 3:**
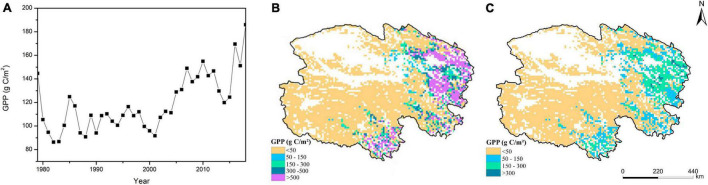
Temporal **(A)** and spatial **(B)** dynamics of GPP and the map of its standard deviation **(C)** in Qinghai grasslands from 1979 to 2018.

The GPP of Qinghai grasslands fluctuated from 1979 to 2018. The annual value fluctuated between 86.33 and 186.00 gC/m^2^, with an average annual value of 118.78 gC/m^2^. After 2,000, GPP increased, and the average annual rate of increase was 4.74 gC/m^2^, indicating that photosynthesis was enhanced during this period (Figure 3A). There were obvious regional differences in GPP and its standard deviation. GPP values in most areas of Qinghai were low, and the high values were mainly distributed in eastern and southern Qinghai (Figure 3B). The spatial distribution of the standard deviation of GPP was generally similar to that of the annual average GPP (absolute values), which indicated that the regions with large interannual change in GPP were mainly concentrated in the regions with high absolute values of the annual averages. On the contrary, the regions with small interannual change in GPP were mainly distributed in the regions with low absolute values of the annual averages (Figure 3C).

**FIGURE 4:**
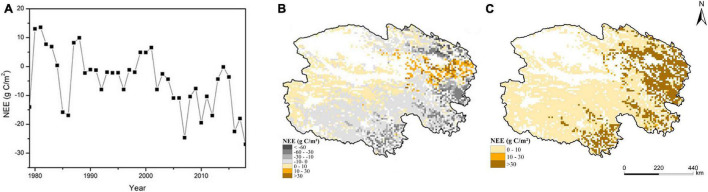
Temporal **(A)** and spatial **(B)** dynamics of NEE and the map of its standard deviation **(C)** in Qinghai grasslands from 1979 to 2018.

The NEE of Qinghai grasslands fluctuated from 1979 to 2018, with an average value of −5.16 gC/m^2^; in most years, NEE values were negative, indicating that this region was a carbon sink. After 2,000, the NEE of Qinghai grasslands decreased in a fluctuating manner, with an average annual rate of decrease of 1.16 gC/m^2^ (Figure 4A). There were pronounced regional differences in NEE and its standard deviation, indicating that the carbon source/sink status varied among areas. The southern, southeastern, and northeastern parts of Qinghai were mainly carbon sinks. The northwestern region and the region between the southeastern and northeastern parts of Qinghai were mainly carbon sources (Figure 4B). The spatial distribution of the standard deviation of NEE was generally similar to that of the annual average NEE (absolute values), which indicated that the regions with large interannual change in NEE were mainly concentrated in the regions with high absolute values of the annual averages. On the contrary, the regions with small interannual change in NEE were mainly distributed in the regions with low absolute values of the annual averages (Figure 4C).

### Effect of Grazing on Gross Primary Productivity and Net Ecosystem Exchange

**FIGURE 5:**
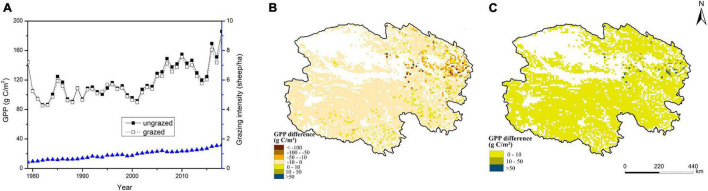
Effect of grazing on the temporal **(A)** and spatial **(B)** dynamics of GPP and the map of the standard deviation of GPP difference **(C)** in Qinghai grasslands from 1979 to 2018 (GPP difference indicates the average annual difference between the grazed and ungrazed scenarios from 1979 to 2018).

Interannual variation in GPP under the grazed scenario was similar to that under the ungrazed scenario in Qinghai grasslands from 1979 to 2018. In general, the GPP under the grazed scenario (average annual value of 115.56 gC/m^2^) was slightly lower than that under the ungrazed scenario, indicating that grazing weakened the photosynthetic capacity of grassland. The difference between the grazed and ungrazed scenarios tended to increase slowly over the study period (Figure 5A). Under the grazed scenario, the GPP values decreased in most areas, and significant decreases in GPP were mainly observed in eastern Qinghai. Under the grazed scenario, significant increases in GPP were mainly observed in southern and eastern Qinghai (Figure 5B). The spatial distribution of the standard deviation of GPP difference between the grazed and ungrazed scenarios was generally similar to that of the annual average GPP difference (absolute values), which indicated that the regions with large interannual change in standard deviation of GPP difference were mainly concentrated in the regions with high absolute values of the annual averages. On the contrary, the regions with small interannual change in standard deviation of GPP difference were mainly distributed in the regions with low absolute values of the annual averages (Figure 5C).

**FIGURE 6:**
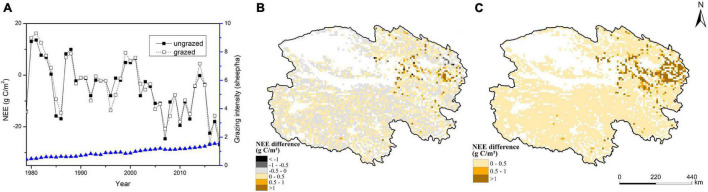
Effect of grazing on the temporal **(A)** and spatial **(B)** dynamics of NEE and the map of the standard deviation of NEE difference **(C)** in Qinghai grasslands from 1979 to 2018 (NEE difference indicates the average annual difference between the grazed and ungrazed scenarios from 1979 to 2018).

From 1979 to 2018, interannual variation in NEE under the grazed scenario was similar to that under the ungrazed scenario. In general, NEE under the grazed scenario fluctuated around the average annual value of −4.64 gC/m^2^, which was slightly higher than that under the ungrazed scenario (−5.05 gC/m^2^), indicating that grazing weakened the carbon sink function of Qinghai grasslands. Under the grazed scenario, the average annual carbon sequestration was 2.14 Tg C in Qinghai grasslands from 1979 to 2018. Under the ungrazed scenario, the average annual fixed carbon was 2.33 Tg C from 1979 to 2018. Grazing decreased carbon fixation by 7.6 Tg C in Qinghai grasslands from 1979 to 2018 (Figure 6A). There were regional differences in the effect of grazing on NEE in Qinghai grasslands. NEE was most strongly affected by grazing in eastern Qinghai (Figure 6B). The spatial distribution of the standard deviation of NEE difference between the grazed and ungrazed scenarios was generally similar to that of the annual average NEE difference (absolute values), which indicated that the regions with large interannual change in standard deviation of NEE difference were mainly concentrated in the regions with high absolute values of the annual averages. On the contrary, the regions with small interannual change in standard deviation of NEE difference were mainly distributed in the regions with low absolute values of the annual averages (Figure 6C).

## Discussion

### Temporal and Spatial Dynamics of Gross Primary Productivity and Net Ecosystem Exchange and the Responses to Grazing

Our study indicated that GPP and NEE fluctuated in Qinghai grasslands from 1979 to 2018 in response to changes in climate. The GPP of grasslands increased and NEE decreased in Qinghai from 2001 to 2018, indicating that the carbon sink capacity was enhanced under the climate change scenario. This was consistent with the results of previous studies (Sun et al., 2016; Lin et al., 2017; You et al., 2019). Our results showed that grasslands in Qinghai generally acted as carbon sinks from 1979 to 2018, which may be associated with the low temperature in most areas of Qinghai grasslands (Figure 1C). Due to the high altitude in Qinghai, the temperature is relatively low, and this decreases the rate of decomposition of organic matter, which promotes carbon accumulation (Lin et al., 2017; Song et al., 2020). Spatial heterogeneity in GPP and NEE was driven by spatial differences in climatic conditions (Figures 1C,D). In general, the hydrothermal conditions were favorable in eastern and southern Qinghai; thus, the photosynthetic capacity and carbon sink capacity were strong for most of the grasslands in this area (Huang et al., 2018; Wang et al., 2019). The opposite pattern was observed in other areas. The low temperature in the southwestern portion of Qinghai weakened the photosynthetic capacity and carbon sink capacity (Figure 1C). Low precipitation in northwestern Qinghai weakened the photosynthetic capacity and carbon sink capacity (Figure 1D; Zhu et al., 2016; Xu et al., 2019). The carbon sink capacity in the southwestern portion of Qinghai was slightly stronger than that in the northwestern portion of Qinghai, which mainly stemmed from differences in temperature. The temperature in the southwestern portion of Qinghai was lower than that in the northwestern portion of Qinghai; this caused the rate of organic matter decomposition to be lower in the southwestern portion of Qinghai, which was more conducive to carbon accumulation (Figure 1C; Lagomarsino et al., 2008; Gomez-Casanovas et al., 2012). GPP was high in the area between the southeastern and northeastern parts of Qinghai. However, the grassland ecosystem in this area is a strong carbon source. This observation might be related to the high level of respiration associated with the high temperature in this area (Dubbert et al., 2014; Peng et al., 2017).

Grazing is the most important use of grasslands in Qinghai, and it has an important impact on the local carbon budget. In this study, the effects of grazing on the grassland ecosystem carbon budget were analyzed by comparing simulation results between grazed and ungrazed scenarios in Qinghai from 1979 to 2018 using the improved Biome-BGCMuSo model. Grazing generally weakened the photosynthetic capacity and carbon sink function of Qinghai grasslands from 1979 to 2018. This was consistent with the widespread unrestricted grazing activities in Qinghai that took place during these years (Dong et al., 2015; Abdalla et al., 2018; Dong S. K. et al., 2020). In general, the GPP under the grazed scenario was slightly lower than that under the ungrazed scenario, and the difference in GPP between these two scenarios tended to increase slowly over the study period, indicating that the extent of excessive grazing was increasing, which is inconsistent with the suggestions of previous studies (Sun et al., 2016; Xu et al., 2016; Wei et al., 2019). These previous studies have shown that ecological restoration driven by the local government coupled with the implementation of effective management measures has greatly improved the ecological environment of Qinghai grasslands. However, in our study, we found that GPP under the grazed and ungrazed scenarios both increased in volatility, and NEE under the grazed and ungrazed scenarios both decreased in a fluctuating manner from 2001 to 2018. Thus, we can infer that climate change, not management, was the main driver of the improvement of the ecological environment of Qinghai grasslands in recent years. In some years (including 1979, 1987–1989, 1992, 1996–1997, 2003, 2005–2006, 2008, and 2015–2016), NEE was lower under the grazed scenario than under the ungrazed scenario, which indicated that grazing generally enhanced the carbon sink function of grasslands in these years. The difference between the NEE values under the grazed and ungrazed scenarios varied depending on the interaction between grass and livestock. Grass growth is closely related to climatic conditions (Zhang et al., 2015; Huang et al., 2018). Thus, the effect of grazing on the carbon sink function depends not only on grazing management but also on climate conditions.

There was pronounced spatial heterogeneity in the effect of grazing on the carbon budget in Qinghai grasslands because of spatial heterogeneity in grazing management and climate conditions. In general, GPP and NEE values were more strongly affected by grazing in eastern Qinghai, which was consistent with the high grass yield (stemming from the favorable hydrothermal conditions) and high grazing intensity in this area (Figure 1B; Huang et al., 2018; Wang et al., 2019). In central and western Qinghai, unfavorable hydrothermal conditions led to low grass yield and low grazing intensity, which was consistent with the small changes in GPP and NEE values observed under the grazed scenario relative to the ungrazed scenario (Figures 5B, 6B). In some areas, grazing enhanced the photosynthetic capacity and carbon sink function, which helped achieve a grass–livestock balance. This pattern may be closely related to the significant overcompensation of the growth of grass under moderate grazing (Huang et al., 2018; Mipam et al., 2019). The opposite pattern was observed in most other areas.

The carbon budget is closely related to variation in climate and grazing management regime in Qinghai grasslands. Appropriate levels of grazing can enhance the photosynthetic capacity and carbon sink function of grasslands. To achieve a balance between grass and livestock, suitable grazing management measures should be implemented depending on the local conditions because of dynamic temporal and spatial variation in climate.

### Comparison With Other Studies

Previous studies of the grassland carbon budget have been conducted in Qinghai grasslands. For example, Zhang et al. (2012) found that the alpine meadow grassland ecosystem acted as a carbon sink on the northern shore of Qinghai Lake using the eddy covariance method. Wu et al. (2018) found that the alpine wet meadow ecosystem around Qinghai Lake behaved as a carbon source using the eddy covariance method. Sun et al. (2019) found that the alpine meadow ecosystem in Arou, Yakou, and Dashalong was a carbon sink using the eddy covariance method. He et al. (2019) found that *Elymus nutans* grassland was a weak carbon sink in a pasture in Tongde County using the eddy covariance method. Song et al. (2020) found that NEE varied seasonally in a grassland ecosystem at Fenghuoshan catchment using data from an eddy covariance tower and chamber-based measurements. Wang et al. (2020) found that an alpine meadow ecosystem was a strong and consistent carbon sink based on a continuous eddy covariance dataset from the Arou super-station. Zhu et al. (2021) found that the alpine meadow at Haibei station was a carbon sink during the growing season using the eddy covariance technique. Most previous studies have shown that the Qinghai grasslands are a carbon sink. This was consistent with the results of our study. However, previous studies have been conducted using data from single sites. Although these previous studies contribute to an in-depth understanding of the carbon budget at the site scale, such studies based on sparse sites do not provide insight into the spatiotemporal dynamics of the grassland carbon budget and the response to grazing because of the high degree of spatial heterogeneity in climate change, terrain, and grazing in Qinghai. In our study, a loop program was used to run the Biome-BGCMuSo model in different pixels of Qinghai grasslands to determine GPP and NEE over large areas. The effect of grazing on the carbon budget was also analyzed by comparing the difference between grazed and ungrazed scenarios.

### Uncertainties in Estimates

In this study, GPP and NEE were estimated in Qinghai grasslands from 1979 to 2018 using the Biome-BGCMuSo model. The reliability of the model outputs was validated by comparison with a large set of observational data under both the grazed and ungrazed scenarios. However, some degree of uncertainty is inevitable.

First, all the models are simplifications of reality. Like many other models, the Biome-BGCMuSo model can introduce uncertainty in the outputs of simulated GPP and NEE because it does not consider the full complexity of the grassland carbon cycle. For example, previous studies have shown that the freezing-thawing cycle substantially affects plant carbon fixation. However, few studies have examined this topic. Thus, the underlying interaction between the freezing-thawing cycle and alpine grasslands could not be fully accounted for in the model, which inevitably introduces uncertainty in the results. In the Biome-BGCMuSo model, the study area was assumed to be composed of a single vegetation type, which means that mixed-species scenarios are not considered. However, overgrazing can lead to the invasion of other grass types, including noxious grasses, that can also fix carbon. This may lead to an underestimation of carbon fixation when the Biome-BGCMuSo model is run under an overgrazing scenario. The trampling of livestock also has an effect on topsoil compaction and plant growth. However, the trampling effect was not considered in the model because it has not been quantified in the previous studies, and this inevitably introduces uncertainty in the results (Hidy et al., 2012, 2016).

Second, model inputs are also a source of uncertainty in the results. In this study, the main inputs of the model were meteorological data and grazing data. Although the accuracy of the meteorological input data in our study is higher compared with the existing reanalysis data, their accuracy is still lower than actual observational data, which inevitably results in uncertainty in model outputs (He et al., 2020). Data on the grazing intensity were produced through spatial interpolation by FAO (see text footnote 2), which inevitably introduces some error. Although we corrected the grazing intensity data according to livestock statistics from the local government to ensure their high precision, a robust correction of these data is difficult given the macroscopic nature of the statistical data from the government.

The spatial heterogeneity in the standard deviations of the model predictions indicated that there were obviously regional differences in the accuracy level of the model predictions. Higher standard deviation is associated with lower reliability of mean value. And in this study the spatial distributions of the standard deviations of the model predictions were generally similar to those of their respective annual means(absolute values). Thus, in Qinghai grasslands the regions with relatively low reliability of the model predictions were mainly concentrated in the regions with high absolute values of the annual average predictions. On the contrary, the regions with relatively high reliability of the model predictions were mainly distributed in the regions with low absolute values of the annual average predictions (Figures 3B,C, 4B,C, 5B,C, 6B,C).

## Conclusion

In this study, the spatiotemporal dynamics of the carbon budget and the response to grazing in Qinghai grasslands from 1979 to 2018 were analyzed using the improved Biome-BGCMuSo model. In general, Qinghai grasslands were carbon sinks. After 2,000, GPP increased and NEE decreased in a fluctuating manner in Qinghai grasslands because of climate change. There was spatial heterogeneity in GPP and NEE under the ungrazed scenario because of differences in climate conditions. In general, high GPP and low NEE occurred in areas with favorable hydrothermal conditions, whereas low GPP and high NEE occurred in areas with unfavorable hydrothermal conditions. Grazing generally weakened the photosynthetic capacity and carbon sink capacity in Qinghai grasslands. There was spatial heterogeneity in the effect of grazing on GPP and NEE because of differences in climate conditions and grazing management measures. In general, GPP and NEE values were more strongly affected by grazing in eastern Qinghai, which was consistent with the high grass yield (stemming from the favorable hydrothermal conditions) and high grazing intensity in this area. The results of this study aid our understanding of the mechanism driving variation in the grassland carbon budget and provide data that could be used to aid local grassland management.

## Data Availability Statement

The raw data supporting the conclusions of this article will be made available by the authors, without undue reservation.

## Author Contributions

XH and HZ conceived, performed, and designed the experiments. XH and CC analyzed the data and performed the computational analysis. XH, BY, ZM, and HZ wrote the manuscript. All authors have approved the final version of the manuscript.

## Conflict of Interest

The authors declare that the research was conducted in the absence of any commercial or financial relationships that could be construed as a potential conflict of interest.

## Publisher’s Note

All claims expressed in this article are solely those of the authors and do not necessarily represent those of their affiliated organizations, or those of the publisher, the editors and the reviewers. Any product that may be evaluated in this article, or claim that may be made by its manufacturer, is not guaranteed or endorsed by the publisher.

## Footnotes

1
https://github.com/hollorol/RBBGCMuso

2
http://www.fao.org/AG/againfo/resources/en/glw/home.html

3
http://down.foodmate.net/standard/yulan.php?itemid=82804

4
http://geodata.pku.eud.cn

5
http://www.fao.org/soils-portal/soil-survey/soil-properties/zh/
