# Supplementary material for: Spatiotemporal Dynamics of the Carbon Budget and the Response to Grazing in Qinghai Grasslands
Source: Front Plant Sci. 2022 Jan 7;12:775015. doi: 10.3389/fpls.2021.775015 (PMC8777210; doi:10.3389/fpls.2021.775015)
Supplement: Supplementary file 1 [file Data_Sheet_1.docx]

**Contribution to the field statement**

Estimating the grassland carbon budget is critically important for ensuring that grassland resources are used sustainably. Previous studies have not characterized the spatiotemporal dynamics of the carbon budget and the response to grazing in Qinghai grasslands. Here, a loop program was designed to run the Biome-BGCMuSo model in different pixels of Qinghai grasslands to estimate the gross primary productivity (GPP) and net ecosystem exchange (NEE) in this region from 1979 to 2018. In general, Qinghai grasslands were carbon sinks. After 2000, GPP increased and NEE decreased in a fluctuating manner in Qinghai grasslands because of climate change. In general, high GPP and low NEE occurred in areas with favorable hydrothermal conditions, whereas low GPP and high NEE occurred in areas with unfavorable hydrothermal conditions. Grazing generally weakened the photosynthetic capacity and carbon sink capacity in Qinghai grasslands. In general, GPP and NEE values were more strongly affected by grazing in eastern Qinghai. The results of this study aid our understanding of the mechanism driving variation in the grassland carbon budget and provide data that could be used to aid local grassland management.
